# Understanding antibiotic use for pig farming in Thailand: a qualitative study

**DOI:** 10.1186/s13756-020-00865-9

**Published:** 2021-01-06

**Authors:** Angkana Lekagul, Viroj Tangcharoensathien, Marco Liverani, Anne Mills, Jonathan Rushton, Shunmay Yeung

**Affiliations:** 1 London School of Hygiene and Tropical Medicine, London, UK; 2 International Health Policy Programme, Ministry of Public Health, Nonthaburi, Thailand; 3 School of Tropical Medicine and Global Health, Nagasaki University, Nagasaki, Japan; 4 Faculty of Public Health, Mahidol University, Bangkok, Thailand; 5 Institute of Infection, Veterinary and Ecological Sciences, University of Liverpool, Liverpool, UK

## Abstract

**Background:**

Antimicrobial resistance (AMR), recognised as a serious and growing threat to global health, is promoted by multiple drivers, including antibiotic use in the livestock sector. Thus, understanding factors influencing antibiotic use in livestock production is essential to the design and implementation of effective interventions to reduce AMR. This qualitative study aimed to explore the experiences and views of the key actors associated with the use of antibiotics for pig farming in Thailand, from local farmers to officers in central government institutions.

**Methods:**

A total of 31 in-depth interviews were conducted with different categories of actors: pig farmers (n = 13), drug retailers (n = 5), veterinarians (n = 7), government officers (n = 3) and representatives of animal and human health associations (n = 2). Themes emerging from the interviews were identified and explored using thematic analysis. In addition, direct observations were conducted in the pig farms.

**Results:**

The findings highlight the multi-faceted nature of the views and practices that may contribute to misuse or overuse of antibiotics in the study locations, including misconceptions about the nature of antibiotics and AMR (particularly among smallholders), lack of facilities and financial means to establish an antibiotic-free farm, lack of sufficient training on AMR and antibiotic prescribing for veterinarians, the profit motive of pharmaceutical companies and their ties to farm consultants, and lack of sufficient regulatory oversight.

**Conclusions:**

Our study indicates a clear need to improve antibiotic use for pig production in Thailand. Farmers need better access to veterinary services and reliable information about animal health needs and antibiotics. Innovative investments in biosecurity could improve farm management and decrease reliance on antibiotics, although the cost of these interventions should be low to ensure wide uptake in the livestock sector. Lastly, further development of professional training and clinical guidelines, and the establishment of a code of conduct, would help improve antibiotic dispensing practices.

## Introduction

Antimicrobial resistance (AMR), recognised as a serious and growing threat to global health, is driven by many factors including antibiotic use not only in humans but also in animals. In many countries, antibiotics are widely applied to promote growth in livestock in addition to preventing and treating infections [1]. This practice has potential risks to human health that need to be addressed [2–4]. Of great concern is the emergence of resistance to those antibiotics categorised by the WHO as Critically Important Antimicrobials (CIA), such as colistin, which are reserved for treating the most severe human infections [5].

In the pig sector, intensive use of antibiotics has promoted resistance of both commensal and pathogenic bacteria [6, 7], particularly in low- and middle-income countries (LMICs) [8, 9]. In view of this, research efforts have been made to explore the factors influencing antibiotic use in pig farms [10–16]. A recent systematic review showed that antibiotics are commonly used during the suckling and post-weaning stages of production; in addition, the same review found that specific farm characteristics (such as the density of pigs) influence the use of antibiotics [16]. Apart from the factors associated with pig production, knowledge and understanding of antibiotics among farmers are also important. A number of studies found that farmers may have limited knowledge of the names of antibiotics and their correct usage [10, 12]. For example, a study in China found an association between poor knowledge of antibiotics and inappropriate use in the farms [11]. Findings about the impact of legislation and government policies on antibiotic use have been mixed. In five European countries, farmers were worried about the implications of legal provisions to reduce antibiotic use, particularly their impact on farm maintenance and costs [13]. In two other surveys in Europe, legislation regarding veterinary drugs was perceived to influence prescribing practices more than the price of antibiotics, market demand or clinical guidelines [14, 15].

Despite these studies, our knowledge of practices influencing the agricultural use of antibiotics is still scarce, especially in countries where resources to conduct research and evaluation are more limited. Considering this gap in knowledge, this article reports findings from a study which aimed to explore the experiences and views of key *actors* associated with the use of antibiotics for pig farming in Thailand, from local farmers to officers in central government institutions. After a description of the study context, methods, and the presentation of findings, implications of the study for the design and implementation of action plans on AMR are discussed.

## Materials and methods

### Study context

The Thai agricultural sector accounts for approximately 10% of GDP (USD 42 billion in 2018) with livestock production, including pigs, contributing around USD 400 million [17]. In 2017, nearly 19.5 million pigs were raised and slaughtered, mainly for the domestic market [18, 19]. Since the 1960s, pig production in the country has increasingly shifted from smallholder farming for household consumption to intensive commercial production for the growing urban markets. The pig sector is dominated by a small number of large agro-industrial conglomerates although a diversity of production systems coexist [20], characterised by different levels of bio-security [21]. In smallholder farms, pigs receive a variety of feed including leftover food and vegetables. Such farms have often limited access to veterinary services and antibiotics, while in commercial farms antibiotics are usually applied to whole groups of pigs through medicated feed, either commercial or mixed in the farm. In 2017, it was estimated that about 3,690 tonnes of antibiotics were given to food-producing animals, of which about 50% belonged to the CIA group [19]. To improve farm management, the Thai Department of Livestock Development (DLD) grants Good Agriculture Practices (GAP) certificates to farms which comply with standards of animal husbandry [22]. GAP-certified farms are required to have designated veterinarians to supervise the control, prevention and treatment of animal diseases, including the use of antibiotics. GAP certification is voluntary.

### Study design

This qualitative study was conducted between March 2018 and January 2019 in a province in the central region of Thailand, which accounts for about 20% of annual domestic pig production and hosts different production systems, from smallholders to large industrial farms. The study was part of a larger project which included a cross-sectional survey of antibiotic use among pig farmers in six sub-districts with the highest number of pig farms in the same province [23]. The research design for the qualitative study was meant to capture the diversity of actors in the pig farming sector that may influence antibiotic use at different level of analysis, from disease prevention and control in the farms to the wider regulatory environment. In practice, data collection primarily involved interviews with farmers to explore their views and practices related to antibiotic use. In parallel with the interviews with farmers, observations were conducted to gain a better understanding of management practices in the same farms. In order to capture the diversity of perspectives, interests, and incentives which may influence antibiotic use, veterinarians, drug retailers, industry representatives, and government officers were also interviewed.

### Participant selection

Participants in this study were recruited from the larger sample of 84 farmers included in the cross-sectional survey [23]. In total, 11 out of the 84 farmers agreed to participate in the study reported here. Two farmers who did not use antibiotics were purposively selected through a snowball sampling technique. In addition, informants who could provide further insight into the use of antibiotics for pig production were approached at relevant organisations, including government offices, the Thai Feed Mill Association, and associations of human and animal health professionals. The first author contacted potential informants to ask if they were able and willing to participate in the study.

### Data collection

Drawing from previous studies [10, 24], the guidelines for the interviews with farmers covered: (a) animal health and farm management, (b) pig production and market demand, (c) relationships with other farmers, veterinarians, pharmaceutical companies, and (d) regulation and policy on antibiotic use (see Additional file 1). Interviews with other categories of participants were tailored to their role and the expertise they could bring to this study. Interviews were conducted face-to-face by the first author and lightly structured to let participants express their own views. Interviews were conducted either in the farms or the offices or shops of key informants. On average, interviews lasted two hours. Written field notes were taken and, where permission was given, the interview was audio-recorded. After the interviews with pig farmers, the researcher sought permission to conduct observations in their farm. During the observations, the researcher examined activities of farm workers, the feed labels, the medicines used in the farm, and general sanitation and farm management practices. In addition, the researcher walked through the farms and engaged in casual conversations with farmers and farm workers. To prevent cross-infection between farms, farm visits were restricted to no more than one a week.

### Data processing and analysis

The interview audio recordings were transcribed verbatim and anonymised by the researcher (AL). Data were imported into the software NVivo 12 for qualitative analysis. The researcher (AL) generated initial codes after iterative reading of the transcripts. The field notes were reviewed in parallel with the transcripts. Then two researchers (AL and VT) identified and organised themes and sub-themes. To reduce subjective bias, the researchers (AL, ML, SY and VT) discussed emerging findings and their interpretation throughout the process of analysis. In qualitative data analysis, themes are considered robust when they are cohesive and meaningful within the entire data set [25]. Thus, consistency both within the individual interviews and across respondents by triangulation was assessed.

## Results

### Profiles of participants and studied farms

Table 1 shows the profiles of the 31 participants interviewed, which consisted of farmers, animal drug retailers, veterinarians, and informants at government offices or relevant professional associations. Table 2 shows the characteristics of the 13 farms, which ranged from a smallholder farm with only one sow and five piglets to a large commercial farm with more than 10,000 pigs and a monthly income of more than US$15,900. Six farms were DLD GAP-certified, one was a contracted farm and five farms were members of a cooperative. Three were fattening farms and ten were farrow-to-finish farms. Two farms were antibiotic-free. Research observations were allowed in six farms with variable characteristics, including “backyard” production and large commercial farms.

**Table 1:** Respondents’ profiles

	Total	Gender		Age (years; mean, range)	Work experience (years; mean, range)
		Male	Female		
1. Pig farmers	13	10	3	47.9 (35–66)	22.7 (5–50)
2. Animal drug retailers	5	3	2	40.8 (30–48)	15.1 (3.5–24)
3. Veterinarians	8	5	3	49 (31–61)	22.8 (5–37)
4. Government officers	3	2	1	37.3 (31–50)	10 (4–20)
5. Representatives of health and animal professional associations	2	1	1	62.5 (60–65)	10 (8–12)
Total	31	21	10	47.8 (30–66)	16.1 (3.5–50)

**Table 2:** Farm characteristics

	Size of farm	Number of pigs		GAP farm	Contracted farm	Member of coopera-tive	Type of farm	Income from selling pigs per month (US$ 1 = 31.5 THB)	Use of anti-biotics	Farm visit
		Number of sows	Number of other pigs							
1	Smallholder	1	5	N	N	N	FtoF	< US$317	Y	Y
2	Smallholder	5	25	N	N	N	FtoF	< US$317	Y	N
3	Smallholder	4	12	N	N	N	FtoF	< US$317	Y	N
4	Commercial (S)	0	60	N	Y	N	Fattening	US$317–1590	Y	Y
5	Commercial (S)	10	90	N	N	N	FtoF	US$3,170–15,900	Y	N
6	Commercial (S)	40	195	N	N	Y	FtoF	Not willing to respond	Y	N
7	Commercial (S)	0	500	Y	N	Y	Fattening	Not willing to respond	Y	N
8	Commercial (S)	50	200	Y	N	N	FtoF	US$3,170–15,900	N	Y
9	Commercial (M)	140	600	Y	N	N	FtoF	US$3,170–15,900	Y	Y
10	Commercial (M)	600	3000	Y	N	Y	FtoF	US$3170–15,900	Y	N
11	Commercial (M)	0	5000	Y	N	N	Fattening	US$3170–15,900	N	Y
12	Commercial (L)	2000	> 10,000	N	N	Y	FtoF	> US$15,900	Y	Y
13	Commercial (L)	2500	> 10,000	Y	N	Y	FtoF	> US$15,900	Y	N

*GAP* good agriculture practice, *FtoF* farrow-to-finish farm

### Use of antibiotics in pig production: views and experiences of different actors

The analysis of the interviews revealed diverse and at times competing views of different actors in the agricultural sector about antibiotic use – the pig farmers, health professionals, and the pharmaceutical industry, considered in turn in the sections below.

### Pig farmers

#### Perceived health benefits and economic value of antibiotics

All the pig farmers interviewed believed that some form of medication, including antibiotics, was necessary to maintain animal health, and control and prevent disease.

Medicines are really important in my farm. Without medicines, my pigs would be very ill. Antibiotics protect my pigs from becoming worse. [Fs02, female, > 40 years old, non-GAP farm]

At the suckling and nursery stages, the piglets are so vulnerable. I usually apply antibiotics to 100% of them. Whether or not they are sick, I must use antibiotics for prevention… [Fc07, male, > 50 years old, GAP-certified farm]

Many farmers explained that antibiotics are an affordable approach to reduce pig mortality. One farmer estimated that medicated feed cost only 2.7% more than non-medicated feed and administering antibiotics to the whole herd via medicated feed was less labour intensive than individual treatment.

I think that antibiotic use is a cheap solution…and affordable… The cost of production is not really different whether we add medicine [antibiotics] or not. For example, now the cheapest medicine is chlortetracycline. For nursery pigs, the feed mixed with chlortetracycline is baht 22.60 compared with baht 22 per kilogram of regular feed [without antibiotics]. It doesn't add much to my budget. [Fc12, male, > 30 years old, non-GAP farm]

#### Pig farmers’ knowledge of antibiotics and awareness of AMR

Knowledge of antibiotics differed greatly amongst pig farmers. None of the three smallholder farmers understood the word “antibiotics” *(yaa-pati-cheewana)* while commercial farmers could generally differentiate between antibiotics and other medicines. Most farmers who understood the meaning of antibiotics said they used them according to the indications on the package labels or following the recommendations of pharmacists. However, some farmers routinely used high potency antibiotics without clinical justification:

For the treatment of common diseases, I apply a broad-spectrum antibiotic such as amoxycillin. If there is no improvement, I will change to cepha(losporin), cefo(xamine) or enrofloxacin…I believe in higher potency antibiotics. If there is no price difference, I always select higher potency antibiotics [Fc12, male, > 30 years old, non-GAP certified farm]

Commercial farmers also understood the concept of antibiotic resistance but they were elusive when the researcher raised the issue that excessive antibiotic use in the farm was an important contributing factor:

Our pigs are good, clean. I know resistant pathogens, but I don’t think that we (farmers) are involved in it. [Fs03, male, > 40 years old, non-GAP certified farm]

#### Farm management

All interviewed farmers agreed that sound farm management was key to animal health and consequently to reducing the need for antibiotics.

I give more attention to prevention than treatment. Water quality, low pig density and good air ventilation are essential for healthy pigs…. When the pigs are healthy, I don’t need to use antibiotics. [Fc09, female, 45 years old, GAP farm]

**Fig. 1:**
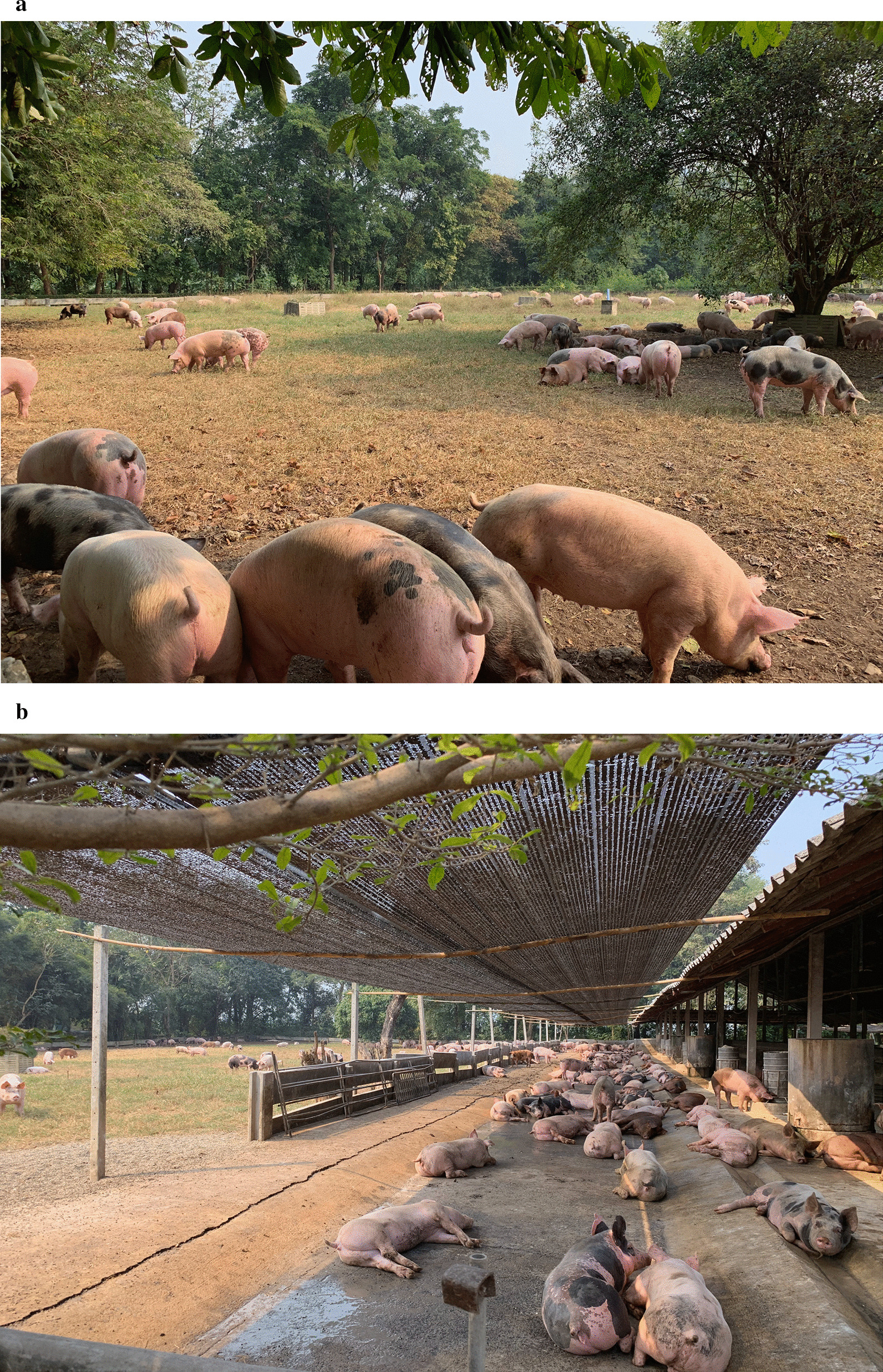
Pigs in the outdoor area at the antibiotic free farm

The government officers in this study also believed that GAP certification contributes to the optimised use of antibiotics. Indeed, the antibiotic-free farms in our study were GAP-certified farms with bio-security measures such as change of clothing and boots and disinfection of all vehicles before entering the farm (Fig. 1). However, only six of the farms in the study were GAP certified. Some farmers were concerned that improving infrastructure and biosecurity to meet GAP standards would require large financial investments.

A closed system housing of 300 m^2^ costs more than 1 million THB (US$ 31,700) …the closed system would improve the health of my pigs and minimise the introduction of pathogens in the farm… so it would reduce the need for antibiotics. But this adds to the production cost. I can't afford it. [Fc06, male, 40 years old, non-GAP-certified farm]

Limited availability of farm veterinarians and gaps in the monitoring system were seen as further challenges to the implementation of GAP requirements.

The GAP criteria are quite strict. They require a farm veterinarian to monitor antibiotic use in the farm. If antibiotic residue is found in pork products, the farm veterinarian must take responsibility. When the farm veterinarian is not available, farmers often give antibiotics to their animals without prescription. [GO3, male, 31 years old].

With the GAP certification, the farmer must report on administrative records all medicines used in the farm and declare they were prescribed by the farm veterinarian. However, farmers may choose to not follow veterinarian’s prescription. We cannot really monitor this. [GO2, female, 50 years old]

Textbox 1: antibiotic-free farm The antibiotic-free farm A was a 5000-fattener farm covering 8,000 m^2^. Eight barns were lined up in an east–west direction to minimize direct sunlight. Each barn had an indoor area of 400 m^2^, with access to an outdoor area of 800 m^2^ for two to three hours during the day, allowing the pigs to move in and out freely. There were no inside partitions, so all 400 pigs (per barn) could live together. The barn floors were made of concrete and cleaned daily by farm workers. Weaning pigs were sourced from another farm, located 17 kms away and owned by the same farmer. If sick pigs were found, they were isolated for treatment or culling.

Observations found that all barns were clean. The researchers also observed pigs roaming around, digging up the ground in search for roots, and also eating fruits from trees that the farmer grew outside the barn. The owner said this farming concept was intended to raise “happy and healthy pigs” because the animals could express their natural behaviour and were not stressed. He said this was a feasible alternative to using antibiotics. The farm received many visitors, with walls lined with photos of international guests and actors.

**Lack of market demand and production facilities for antibiotic-free pork**

The owner of an antibiotic-free farm expressed concerns that market demand for antibiotic-free pork was still low. Another participant pointed out that antibiotic-free standards cannot be fully met in Thailand since most slaughterhouses do not have facilities to separate antibiotic-free pork and medicated pork, causing possible contamination. However, a farmer explained that large companies would not face this problem since they usually control the whole supply chain, including the farm, the slaughterhouse and the retail outlet.

### Health professionals

#### Veterinary services

Antibiotics and other medicines used in the farms were provided by different categories of actors working in the agricultural sector, including veterinary practitioners, veterinarians in pharmacies, representatives of pharmaceutical companies and animal husbandry specialists. Most farmers in our sample relied on the advice of veterinarians regarding the selection and use of antibiotics. However, only one out of 13 farms hired a full-time licensed veterinarian, while the others hired “farm consultants” who were academics, reportedly tied to pharmaceutical companies. Smallholder farmers had limited access to veterinary services, due to lack of public veterinary health facilities and district veterinarians, while most animal clinics served companion animals only. All smallholder farmers received advice on antibiotic use from other farmers or the pharmacies where they purchased antibiotics.

#### Training

Interviews with key informants from the veterinary and pharmacy councils confirmed that courses on the prudent use of antibiotics were not included in the veterinary and animal husbandry curriculum, while the pharmacy curriculum did not cover use of antibiotics in animals. A key informant from a veterinary association mentioned that their association provided in-service training and clinical practice guidelines for disease management. However, veterinarians expressed concern over lack of clinical guidelines, lack of protocols for sample collection, difficulties in laboratory sample transportation, delays in receiving lab results and high cost of bacterial culture and drug sensitivity testing.

#### Awareness of AMR

Most veterinarians were aware of government policy on reducing the use of antibiotics. However, some of them became defensive when the researcher raised the issue of AMR. They said antibiotics were used only when necessary, and not indiscriminately as perceived by the public.

Of course, we use a large amount of antibiotics in livestock, but I believe that other sectors such as doctors, pharmacists and orchards use more. Patients who don't take the full dose are the cause of the resistant bacteria … I don’t believe that people will die from AMR transmitted by animals. [V07, male, 52 years old]]

### The pharmaceutical industry

#### Antibiotic sales and advertisement

Commercial farmers explained they could buy antibiotics easily at pharmacies or from representatives of pharmaceutical companies who visited their farms. Respondents from the three commercial farms also reported that representatives of pharmaceutical companies encouraged the purchase of antibiotics and other medicines through discounts and gifts such as leisure travel.

All pharmaceutical companies offer sales promotions. You can choose either 10% discount or international leisure travel awards. In previous years, I have travelled to the US, Iceland, Spain, Japan. I feel like I have to order more medicines to gain the award. [Fc13, male, > 40 years old, GAP certified farm]

Representatives of pharmaceutical companies offered me a dinner or presents such as a liquor… and they asked me to help them achieve their sales target… [Fc10, male, > 40 years old, GAP certified farm]

In addition, government officers noted that indiscriminate sales were difficult to control due to lack of sufficient human resources.

We have only a few inspectors. We cannot inspect all pharmacies in our catchment area, particularly the animal pharmacies. It is not our priority as the governor gives priority to the control of illegal drugs [GO1, male, 37 years old]

#### Relationship between pharmaceutical companies, farmers, and academia

Farmers who hired academic lecturers as farm consultants felt “obligated” to follow their recommendations on various aspects of farm mangement, including advice on the choice of antibiotics. Two farmers believed that academic consultants would receive gifts from the pharmaceutical companies they recommended, such as equipment for their faculty or honoraria.

Most lecturers are linked with pharmaceutical companies. They support lecturers by providing equipment to their university (…) When these lecturers come here and recommend to purchase the antibiotics from a company, it is difficult to deny their advice. [Fc10, male, > 40 years old, GAP certified farm]

## Discussion

**Fig. 2:**
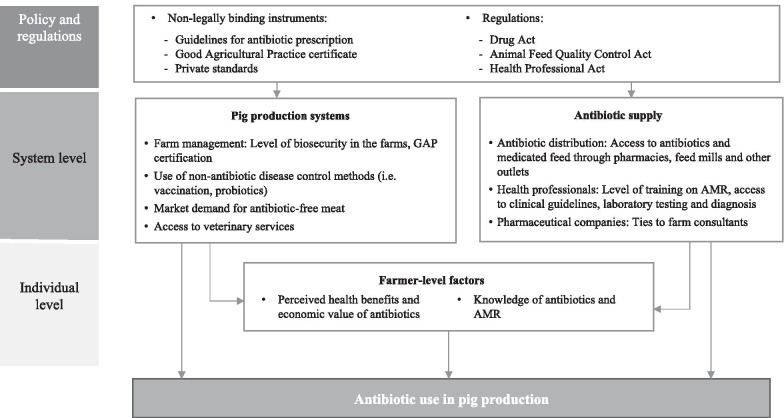
Factors related to the use of antibiotics for pig production in the study locations

This qualitative study aimed to deepen understanding of the complex set of factors influencing the use of antibiotics for pig farming in a particular context of livestock production. As described above and summarised in Fig. 2, the findings highlight the multi-faceted nature of antibiotic use and the complexity of influencing factors, ranging from perceptions (and misunderstandings) about the health benefits of antibiotics to the various interests of the multiple actors involved. A remarkable finding from this study is that many farmers recognised that good farm management practices (such as safe and clean housing and routine vaccination) could greatly reduce disease prevalence and therefore the need for antibiotics. However, only a few farmers could afford the capital investment that is needed to build and maintain an antibiotic-free farm. By contrast, from a farmer’s perspective, intensive use of antibiotics provides a reliable and cost-effective solution to protect animal health and maximise profit. In line with our study, a survey of pig production costs in Spain found that the cost of drugs and vaccines was less than 4.2% of the total [26].

Our findings also showed that farmers, particularly smallholders, may have inadequate understanding of antibiotics and antibiotic resistance. As emerged in some interviews, this can partly be explained by the existence of different ways to refer to “antibiotics” in Thai, including y*aa-kha-chue* (“drug that kills germs”), y*aa-khae-akseab* (“anti-inflammatory drug”) and *yaa-pati-cheewana* (“drug that fights microbes”). The term y*aa-kha-chue* is particularly confusing since it can also be used to indicate other types of drugs such as antifungal, anthelmintic, and antiprotozoal drugs. In addition, *yaa-pati-cheewana* is a technical term which is often used in AMR campaigns but is not commonly understood by lay people as we found in our study. That said, we should bear in mind that a good understanding of antibiotics does not necessarily translate into appropriate use. For example, a study in Lithuania found no correlation between knowledge of antibiotics and their use for self-medication [27]. The cross-sectional survey of 84 pig farmers we conducted as part of this project also found no association between antibiotic understanding and use [23].

Further considering our findings, we can draw some lessons on policy and regulatory issues that need to be addressed to improve antibiotic use in the pig sector. In most countries, it is recognised that veterinarians and animal health authorities should play a key role in providing information about antibiotics and their appropriate use [24, 28]. In Thailand, the Department of Livestock Development (DLD) is mandated to prevent and control animal disease, enforce legal provisions and promote good practices in livestock production [29]. To this end, the DLD relies on a network of farm veterinarians and officers. However, our study suggests that the availability of veterinary services may be insufficient due to gaps in human resources, particularly for smallholders [30]. In addition, lack of effective surveillance systems for infectious diseases in livestock and limited AMR information were perceived to hamper appropriate dispensing of antibiotics by veterinarians. Similarly, a study in European countries found that veterinarians seldom used sensitivity tests to inform decisions about antibiotic use due to the excessive time lag between testing and results [15]. Veterinarians were also found to have business concerns. such as the need to maintain good relationships with clients and the cost of laboratory diagnosis [31], which are not conducive to appropriate antibiotic dispensing [24, 31–33]. These problems are also apparent in the human health sector, where conflicts of interest between healthcare providers and pharmaceutical companies may lead to inappropriate prescribing behaviour and create negative public perceptions towards health professionals [34–37].

Lastly, the role of pharmaceutical companies and market incentives to promote antibiotic use, including the provisions of gifts and other rewards, deserves particular attention [36–38]. In some countries, codes of conduct and ethical guidelines to regulate the behaviour of pharmaceutical companies are in place [39, 40]. In the UK, for example, the Code of Practice for the Promotion of Animal Medicine restricts the advertisement of animal medicines [41]. In Thailand, the production and use of certain veterinary antibiotics was regulated in 2019. Specifically, farmers need a veterinary prescription to produce farm-mixed medicated feed and to use other types of antibiotics in their farms such as injections or medicated water with quinolones and derivatives, cephalosporins, macrolides or polymyxins [42]. However, implementation of this regulation has been slow and compliance is not yet monitored. In addition, there are no codes of conduct or ethical guidelines to regulate advertisement and marketing practices.

In the future, rules on market access could help increase safety standards for the production, processing and sale of pig products [43, 44]. In recent years, private food safety standards have been implemented in Thailand, including those related to antibiotic residue testing in food products and antibiotic-free pork production, particularly in large commercial farms. By law, animals that are given antibiotics cannot be slaughtered until the withdrawal period ends [45] and the maximum residue limit of veterinary drugs in food is set by the Food and Drug Administration [46]. When residue violations are detected, the Thai-FDA or DLD must take legal action against violators and remove the contaminated products from the market.

Yet tighter restrictions on the use of antibiotics may have a negative impact on the financial viability of smallholders. First, a large farm has higher capacity to replace antibiotic use with other preventive measures such as vaccination and improved infrastructure, while smallholders may not be able to afford these additional costs. Second, as we have seen, the pig production and supply chain in Thailand is structured in a way that limits access of smallholder farmers to the premium markets of antibiotic-free products. Large farm owners can produce antibiotic-free pork in their own farms, process the meat in their slaughterhouses and pack the final product in their retail shops, ensuring supply to premium markets from the farm to the fork. In contrast, interviews with farmers revealed that antibiotic-free pork and other pork are not processed and packed separately at external slaughterhouses. As a result, those who cannot afford the maintenance of a slaughterhouse may find it difficult to produce antibiotic-free meat.

### Study limitations

To our knowledge, this is the first qualitative investigation of factors influencing the use of antibiotics in pig production in a middle-income country. The study aimed to unpack the complexity of interactions among actors involved in antibiotic use, in the wider policy and regulatory context. However, limitations should be noted. Since AMR is a sensitive issue in Thailand, the number of farmers who agreed to participate in the study was rather small. In addition, findings cannot be generalised widely although we hope this study can provide useful insights to better understand antibiotic use in many other settings with similar livestock production systems, markets and regulatory environments.

### Policy recommendations

Our study highlights the need to improve antibiotic use for pig production in Thailand. Given that farmers had limited knowledge and awareness of antibiotics and AMR, access to veterinary services and reliable information about animal health needs to be improved, particularly for smallholder farmers. Innovative low-cost investment in biosecurity could result in better farm management leading to effective disease control, improved animal health and decreased reliance on antibiotics. Poor antibiotic prescribing could be addressed through continued professional development and training, stronger undergraduate curricula, and monitoring adherence to clinical guidelines. Controlling the commercial interests of the industry and health professionals in promoting antibiotics will also require the establishment, enforcement and monitoring of a code of conduct. Finally, the combination of private market access rules and control through regulations could be another effective instrument to govern the use of antibiotics where other approaches are ineffective.

## Supplementary Information

**Additional file 1**. Factors influencing the use of antibiotics for pig production in Thailand: Interview guide.

## Availability of data and material

The datasets generated during and/or analysed during the current study are available from the corresponding author upon reasonable request.

## References

[1] World Organisation for Animal Health. The Third OIE Annual report on the use of antimicrobial agents in animals. Paris; 2018. www.oie.int. Cited 20 Aug 2019.

[2] Van Boeckel TP, Glennon EE, Chen D, Gilbert M, Robinson TP, Grenfell BT (2017). Reducing antimicrobial use in food animals. Science (80-).

[3] World Health Organization (2015). Global action plan on antimicrobial resistance.

[4] World Health Organisation. United Nations meeting on antimicrobial resistance. Vol. 94, Bulletin of the World Health Organization. World Health Organization; 2016. https://www.who.int/antimicrobial-resistance/events/UNGA-meeting-amr-sept2016/en/. Cited 4 Feb 2020. 10.2471/BLT.16.020916 PMC5034641 27708467

[5] Liu YY, Wang Y, Walsh TR, Yi LX, Zhang R, Spencer J (2016). Emergence of plasmid-mediated colistin resistance mechanism MCR-1 in animals and human beings in China: a microbiological and molecular biological study. Lancet Infect Dis.

[6] Zhang B, Ku X, Yu X, Sun Q, Wu H, Chen F (2019). Prevalence and antimicrobial susceptibilities of bacterial pathogens in Chinese pig farms from 2013 to 2017. Sci Rep.

[7] Holmer I, Salomonsen CM, Jorsal SE, Astrup LB, Jensen VF, Høg BB (2019). Antibiotic resistance in porcine pathogenic bacteria and relation to antibiotic usage. BMC Vet Res.

[8] Lam Y, Fry JP, Nachman KE (2019). Applying an environmental public health lens to the industrialization of food animal production in ten low- and middle-income countries. Glob Health.

[9] Van Boeckel TP, Brower C, Gilbert M, Grenfell BT, Levin SA, Robinson TP (2015). Global trends in antimicrobial use in food animals. Proc Natl Acad Sci USA.

[10] Om C, McLaws ML (2016). Antibiotics: practice and opinions of Cambodian commercial farmers, animal feed retailers and veterinarians. Antimicrob Resist Infect Control.

[11] Chen XI, Wu L, Xie X. Assessing the linkages between knowledge and use of veterinary antibiotics by pig farmers in rural China. Int J Environ Res Public Health. www.mdpi.com/journal/ijerph. Cited 7 Aug 2019. 10.3390/ijerph15061126 PMC6024981 29857480

[12] Eltayb A, Barakat S, Marrone G, Shaddad S, Stålsby LC (2012). Antibiotic use and resistance in animal farming: a quantitative and qualitative study on knowledge and practices among farmers in Khartoum, Sudan. Zoonoses Public Health.

[13] Visschers VHM, Backhans A, Collineau L, Iten D, Loesken S, Postma M (2015). Perceptions of antimicrobial usage, antimicrobial resistance and policy measures to reduce antimicrobial usage in convenient samples of Belgian, French, German, Swedish and Swiss pig farmers. Prev Vet Med.

[14] De Briyne N, Atkinson J, Pokludová L, Borriello SP, Price S. Factors influencing antibiotic prescribing habits and use of sensitivity testing amongst veterinarians in Europe. 2013. http://veterinaryrecord.bmj.com/content/vetrec/173/19/475.full.pdf. Cited 20 Apr 2018. 10.1136/vr.101454 PMC3841786 24068699

[15] Carmo LP, Nielsen LR, Alban L, da Costa PM, Schüpbach-Regula G, Magouras I (2018). Veterinary expert opinion on potential drivers and opportunities for changing antimicrobial usage practices in livestock in Denmark, Portugal, and Switzerland. Front Vet Sci.

[16] Lekagul A, Tangcharoensathien V, Yeung S (2019). Patterns of antibiotic use in global pig production: a systematic review. Vet Anim Sci.

[17] Office of the National Economic and social Development Board (2018). Gross domestic product chain measures 3/2561.

[18] Department of Livestock Development M of A and C. Number of livestock inventory in Thailand on 2017. Bangkok; 2018. http://en.dld.go.th/index.php/en/statistics-menu/statistics-livestock-in-thailand-menu/190-number-livestock2017. Accessed 30 Oct 2018.

[19] Thai Working Group on Health Policy and Systems Research on Antimicrobial Resistance. Consumption of antimicrobial agents in Thailand in 2017, vol. 1. 2018. www.fda.moph.go.th. Accessed 30 Oct 2018.

[20] Thanapongtharm W, Linard C, Chinson P, Kasemsuwan S, Visser M, Gaughan AE (2016). Spatial analysis and characteristics of pig farming in Thailand. BMC Vet Res.

[21] World Organisation for Animal Health (OIE). Glossary of term. 2019. https://www.oie.int/fileadmin/Home/eng/Health_standards/tahc/current/glossaire.pdf. Cited 26 Nov 2019.

[22] National Bureau of Agricultural Commodity and Food Standards; Ministry of Agriculture and Cooperatives (organization). Good agricultural practices for pig farm. Bangkok; 2009.

[23] Lekagul A, Tangcharoensathien V, Mills A, Rushton J, Yeung S (2020). How antibiotics are used in pig farming: a mixed-methods study of pig farmers, feed mills and veterinarians in Thailand. BMJ Glob Health.

[24] Coyne LA, Latham SM, Williams NJ, Dawson S, Donald IJ, Pearson RB (2016). Understanding the culture of antimicrobial prescribing in agriculture: a qualitative study of UK pig veterinary surgeons. J Antimicrob Chemother.

[25] O’Reilly M, Parker N (2013). “Unsatisfactory Saturation”: a critical exploration of the notion of saturated sample sizes in qualitative research. Qual Res.

[26] Rocadembosch J, Amador J, Bernaus J, Font J, Fraile LJ (2016). Production parameters and pig production cost: temporal evolution 2010–2014. Porc Health Manag.

[27] Pavydė E, Veikutis V, Mačiulienė A, Mačiulis V, Petrikonis K, Stankevičius E (2015). Public knowledge, beliefs and behavior on antibiotic use and self-medication in Lithuania. Int J Environ Res Public Health.

[28] Garforth CJ, Bailey AP, Tranter RB (2013). Farmers’ attitudes to disease risk management in England: a comparative analysis of sheep and pig farmers. Prev Vet Med.

[29] Department of Livestock Development. Vision & Mission, Department of Livestock Development. 2019. http://en.dld.go.th/index.php/en/about-us/vision-mission. Cited 21 Jun 2019.

[30] Ratanakorn P, Moonarmart W, Urkasemsin G, Tiensin T. The relationship between the VSB, Veterinary Services and Veterinary Associations in Thailand. In: Global conference on veterinary education and the role of the veterinary statutory body. Foz de Iguazu, Brazil; 2013.

[31] King C, Smith M, Currie K, Dickson A, Smith F, Davis M (2018). Exploring the behavioural drivers of veterinary surgeon antibiotic prescribing: a qualitative study of companion animal veterinary surgeons in the UK. BMC Vet Res.

[32] Coyne LA, Pinchbeck GL, Williams NJ, Smith RF, Dawson S, Pearson RB (2014). Understanding antimicrobial use and prescribing behaviours by pig veterinary surgeons and farmers: a qualitative study. Vet Rec.

[33] Springer S, Sandøe P, Lund TB, Grimm H (2019). Patients interests first, but … ”–austrian veterinarians’ attitudes to moral challenges in modern small animal practice. Animals.

[34] Blumenthal D (2004). Doctors and drug companies. N Engl J Med.

[35] Orlowski JP, Wateska L (1992). The effects of pharmaceutical firm enticements on physician prescribing patterns; there’s no such thing as a free lunch. Chest.

[36] Studdert DM, Mello MM, Brennan TA (2004). Financial conflicts of interest in physicians’ relationships with the pharmaceutical industry—self-regulation in the shadow of federal prosecution. N Engl J Med.

[37] Zaki NM (2014). Pharmacists’ and physicians’ perception and exposure to drug promotion: a Saudi study. Saudi Pharm J.

[38] Goupil B, Balusson F, Naudet F, Esvan M, Bastian B, Chapron A (2019). Association between gifts from pharmaceutical companies to French general practitioners and their drug prescribing patterns in 2016: retrospective study using the French Transparency in Healthcare and National Health Data System databases. BMJ.

[39] Hernéndez DB, Alberola AM, Bermejo DG, Company ES (2017). The reason for having a code of pharmaceutical ethics: Spanish pharmacists code of ethics. Farm Hosp.

[40] Francer J, Izquierdo J, Music T, Narsai K, Nikidis C, Simmonds H (2014). Ethical pharmaceutical promotion and communications worldwide: codes and regulations. Philos Ethics Humanit Med.

[41] National Office of Animal Health Ltd. Code of practice for the promotion of animal medicines. 2017. https://www.noah.co.uk/wp-content/uploads/2017/11/Code-of-Practice-Booklet-28-effective-December-2017.pdf. Cited 11 Aug 2019.

[42] Ministry of Public Health. Ministry of Public Health Notification No.50. 2019. http://www.fda.moph.go.th/sites/drug/SharedDocuments/Law03-TheMinistryOfHealth/SPC(50).pdf.

[43] Wouters J, Geraets D. Private food standards and the World Trade Organization: some legal considerations. In: World trade review. 2012.

[44] Liu P. Private standards in international trade: issues and opportunities. Geneva; 2009.

[45] Department of Livestock Development Ministry of Agriculture and Cooperatives. Notification of the Department of Livestock Development: medicated feed which not allow to be produced, imported, sold and used. 2018. http://afvc.dld.go.th/index.php/2016-04-12-04-46-53/กฏหมาย/พระราชบัญญัติควบคุมคุณภาพอาหารสัตว์-พ.ศ.2558/ประกาศกระทรวงเกษตรและสหกรณ์/กำหนดลักษณะและเงื่อนไขของอาหารสัตว์ที่ผสมยาที่ห้ามผลิต-นำเข้า-ขาย-และใช้-พ.ศ.2561/.

[46] Ministry of Public Health. Notification of the Ministry of Public Health (No. 303) B.E. 2550 (2007) Re: veterinary drugs residues in foods. 2007.

