# Supplementary material for: Understanding antibiotic use for pig farming in Thailand: a qualitative study
Source: Antimicrob Resist Infect Control. 2021 Jan 6;10:3. doi: 10.1186/s13756-020-00865-9 (PMC7789695; doi:10.1186/s13756-020-00865-9)
Supplement: Supplementary file 1 — Additional file 1. Factors influencing the use of antibiotics for pig production in Thailand: Interview guide. [file 13756_2020_865_MOESM1_ESM.docx]

**Factors influencing the use of antibiotics for pig production in Thailand**

**Interview guide**

**1: Animal health and farm management**

**Sub-topic**: General views and practices related to health management

1. Can you tell me about health management practices in your farm?
2. What is the main health problem(s) in your farm?
3. Do pigs at different production stages have different health issues?
4. If your pigs get sick, how do you treat them? Do you use any medicines? If so, what kind of medicines? Do you use them for the prevention of treatment of diseases?
5. <In case the farmer does not use antibiotics> Why did you decide to not use antibiotics?
6. Are your pigs vaccinated? If so, what kinds of vaccination program do you use?
7. Do you think that vaccination helps reducing antibiotic use?

**Sub-topic**: Farm management and antibiotic use

1. Do you believe that antibiotics are necessary for animal farming? If so, why?
2. Do you think any of these issues may be associated with increased antibiotic use in the farm?

- Poor farm management
- Suboptimal feed quality

1. In this period, is there any disease outbreaks in your farm or other parts of the province?

- If so, does this affect farm management including the use of antibiotics? How?

1. If you work for a larger company, does this company set the standards of health management including antibiotic use? If so, how?

**Area 2: Pig production and market demand**

**Sub-topic**: Pig production and antibiotic use

- In your experience, what is the effect of antibiotics on the morbidity and mortality of pigs?
- Do you think antibiotics are necessary to sustain economic profit?
- What about their cost? Are antibiotics expensive?

**Sub-topic**: food safety, market demand (consumer preference)

- What do you think about antibiotics and food safety? Is there a way to monitor the antibiotic residue in your meat?
- What do you think about consumer’s demand for organic food or antibiotic-free pig products? Do you think this may affect antibiotics use in the farms? If so, how?

**Area 3: Relationships with other farmers, veterinarian, pharmaceutical company**

**Sub-topic: Relations with other farmers**

1. Have you ever talked or shared information about antibiotics with other farmers you know? If so, can you explain?
2. Do you belong to a cooperative association? If so, has this ever involved the discussion or sharing of information about antibiotic use? If so, can you explain?

**Sub-topic: roles of veterinarians in diagnosing disease and prescribing antibiotics**

1. Who does animal health checks and prescribes antibiotics in your farm?

- Do you receive regular visits from farm veterinarians? If so, can you explain?
- Have you ever received visits in your farm from DLD officers? If so, can you explain?
- Do you rely on the service of veterinarian consultants? If so, are they from academia? Which university are they from? Do they influence your decision on the use of antibiotics?

**Sub-topic: Sources of antibiotics**

1. Where do you buy the medicines (including antibiotics) and medicated feed?
2. Can you tell me more about how you purchase them?

- What about sale representatives? Do they try to influence the way you use antibiotics in the farm? If so, how?

**Area 4: Regulation and policy on antibiotic use**

**Sub-topic: Regulations**

1. Do you have an official standard farm certification?
2. Do you receive any inspections from the DLD? If yes, how frequently? What do they monitor?
3. Is there any other organisation inspecting your farm? If yes, who and how?
4. Have you heard about any regulations restricting the use of active pharmaceutical ingredient in animal feed and antibiotic use for growth promotion?
   1. If so, what do you think?
   2. Can regulations be applied easily in your farm?
5. The Thai-FDA and DLD are currently working on a new regulation to optimise the use of antibiotics in livestock to reduce AMR
   1. Do you know this? If so, what do you think?

**Sub-topic: Policy on antibiotic use**

1. Are you aware of the National Strategic Plan on AMR, and the national target to reduce 30% of antibiotic use in animals? If so, what do you think?
